# SCARF-1 promotes adhesion of CD4^+^ T cells to human hepatic sinusoidal endothelium under conditions of shear stress

**DOI:** 10.1038/s41598-017-17928-4

**Published:** 2017-12-14

**Authors:** Daniel A. Patten, Sivesh K. Kamarajah, Joanne M. Rose, Joseph Tickle, Emma L. Shepherd, David H. Adams, Chris J. Weston, Shishir Shetty

**Affiliations:** National Institute for Health Research Birmingham Liver Biomedical Research Unit and Centre for Liver Research, Institute of Immunology and Immunotherapy, University of Birmingham, Birmingham, United Kingdom

## Abstract

Liver-resident cells are constantly exposed to gut-derived antigens via portal blood and, as a consequence, they express a unique repertoire of scavenger receptors. Whilst there is increasing evidence that the gut contributes to chronic inflammatory liver disease, the role of scavenger receptors in regulating liver inflammation remains limited. Here, we describe for the first time the expression of scavenger receptor class F, member 1 (SCARF-1) on hepatic sinusoidal endothelial cells (HSEC). We report that SCARF-1 shows a highly localised expression pattern and co-localised with endothelial markers on sinusoidal endothelium. Analysis of chronically inflamed liver tissue demonstrated accumulation of SCARF-1 at sites of CD4^+^ T cell aggregation. We then studied the regulation and functional role of SCARF-1 in HSEC and showed that SCARF-1 expression by HSEC is regulated by proinflammatory cytokines and bacterial lipopolysaccharide (LPS). Furthermore, SCARF-1 expression by HSEC, induced by proinflammatory and gut-derived factors acts as a novel adhesion molecule, present in adhesive cup structures, that specifically supports CD4^+^ T cells under conditions of physiological shear stress. In conclusion, we show that SCARF-1 contributes to lymphocyte subset adhesion to primary human HSEC and could play an important role in regulating the inflammatory response during chronic liver disease.

## Introduction

The liver receives 75–80% of its blood supply from the gut and consequently the cells of the liver are exposed to a vast array of microbial antigens. In order to cope with this constant antigenic load, liver cells express a range of professional pattern recognition receptors, that allow them to discriminate between harmless and damaging antigens^1^. There is now increasing evidence to implicate these gut-derived, microbial-associated molecular patterns (MAMPs) in contributing to a range of liver diseases including non-alcoholic fatty liver disease (NAFLD), alcoholic liver disease (ALD) and autoimmune liver diseases, such as primary biliary cholangitis (PBC) and primary sclerosing cholangitis (PSC)^2^. Thus far, research has focused on TLRs as key players in the innate immune response to MAMPs^3^ but other classes of pattern recognition receptors are also likely to play an important role.

Scavenger receptors are a large superfamily of proteins first identified by their ability to bind and subsequently internalise oxidised low density lipoproteins (oxLDLs)^4^. They are now known to bind multiple endogenous and exogenous products^5^, including a wide array of microbial antigens^6^. Functionally, scavenger receptors play important roles in the maintenance of tissue homeostasis and protection from infection but they may also be implicated in the persistence of injury in inflammatory disorders including chronic liver diseases^5,7^. Scavenger receptors expressed by hepatocytes and resident macrophages (Kupffer cells) have been implicated in the pathogenesis of viral hepatitis^8,9^, metabolic-induced liver injury^10,11^ and fibrosis^12,13^. Hepatic sinusoidal endothelial cells (HSEC), which represent the second most abundant cell type in the human liver, express an array of scavenger receptors at high density consistent with their role in removing microbial antigens from the portal blood. We have also reported that they play an important role in leukocyte recruitment to the liver. Previous work has shown that the scavenger receptor, Stabilin-1, is expressed by HSEC in a range of chronic liver diseases and hepatocellular carcinoma^14,15^ where it is involved in the recruitment of regulatory T (T_reg_) lymphocytes and B cells to the liver^15,16^. Additionally, the Stabilin-1 homologue, Stabilin-2^17^, CD36^18^ and scavenger receptor BI^19^ have also been reported to be expressed in HSEC.

Scavenger receptor class F, member 1 (SCARF-1), also known as scavenger receptor expressed by endothelial cells (SREC)-I is expressed in murine liver sinusoidal endothelial cells^20^; however, its cell-specific expression and function in the human liver is unknown. SCARF-1 is an evolutionarily conserved scavenger receptor^21^, first identified in cDNA libraries from human umbilical vein endothelial cells (HUVEC)^22^. SCARF-1 has been shown to bind modified low density lipoproteins (LDLs), specifically acetylated-LDLs (acLDLs)^23^, and acts as an endocytic receptor for a wide range of damage-associated products including heat-shock proteins (Hsps)^24–26^ and apoptotic host cells via the C1q protein^27^. In addition to binding and internalising a diverse range of endogenous proteins, SCARF-1 also binds a wide array of viral^20,28,29^, fungal^21^ and bacterial^30–33^ antigens. SCARF-2, also known as SREC-II, shows a 35% homology to SCARF-1 and exhibits a similar transcriptional expression pattern across a range of human tissues^34^; however, less is known about the scavenging function of SCARF-2, with SCARF-1 being its only known ligand^34^.

In this study, we describe SCARF-1 expression in the sinusoids and major vessels of the normal human liver and within fibrotic septa of chronic liver diseases and the peritumoral stroma of hepatocellular carcinoma (HCC). In view of the sinusoidal and vascular pattern of SCARF-1 expression we hypothesised that it may have a role in leukocyte recruitment. Initially, we detected SCARF-1 expression in isolated HSEC and showed its up-regulation *in vitro* by proinflammatory cytokines, bacterial LPS and tumourigenic growth factors. Functionally, we demonstrate that immobilised recombinant human (rh)SCARF-1 can directly interact with CD4^+^ T lymphocytes in the presence of vascular cell adhesion molecule (VCAM)-1 *in vitro*, and can support CD4^+^ T cell adhesion to HSEC stimulated with TNFα and LPS, under conditions of physiological flow through a SCARF-1 rich adhesive cup-like structure containing adherent CD4^+^ T cells.

## Results

### SCARF-1 in normal liver, chronic liver disease and malignancy

**Figure 1:**
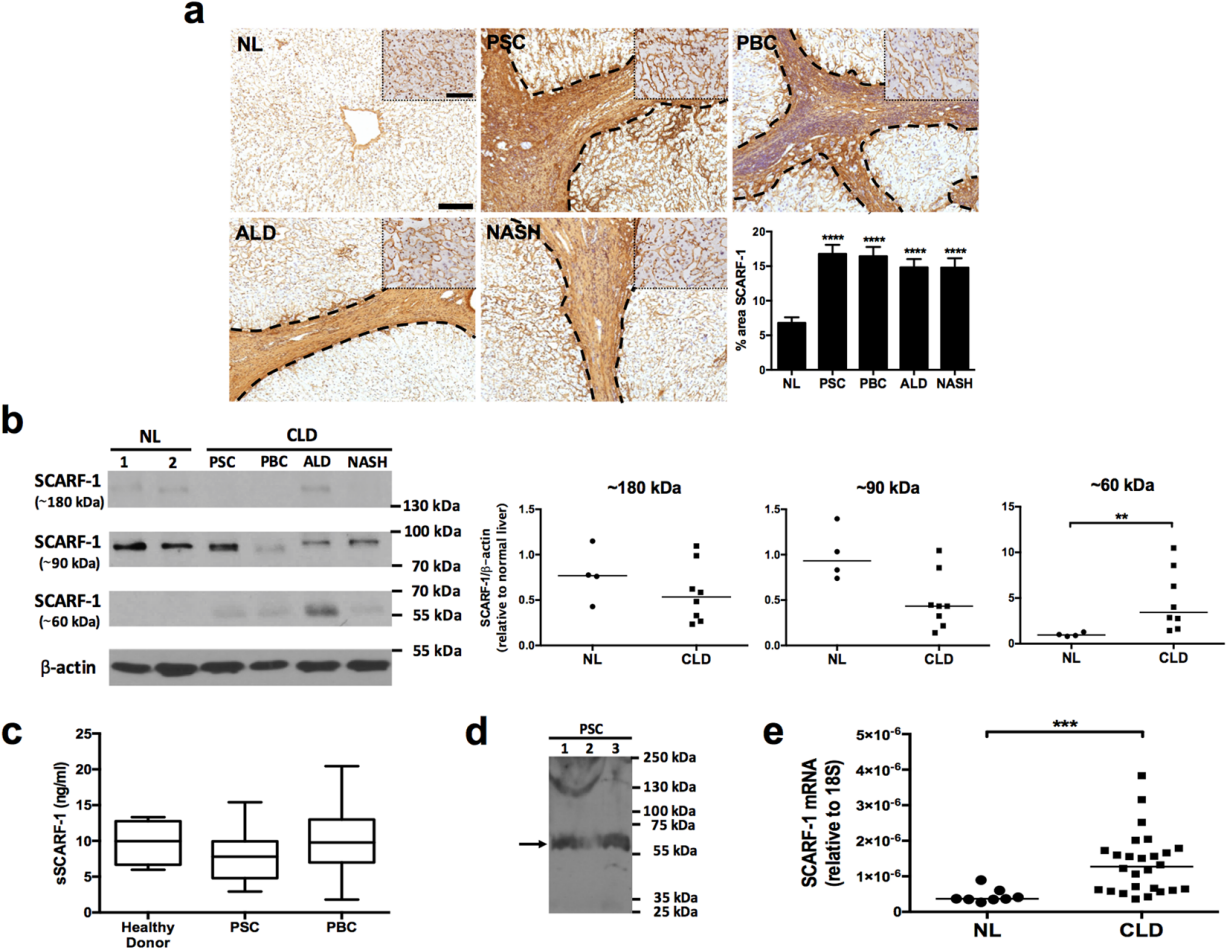
SCARF-1 expression is up-regulated in chronic liver disease. (**a**) Immunohistochemical staining of SCARF-1 (brown) in representative images of normal liver (NL), primary sclerosing cholangitis (PSC), primary biliary cholangitis (PBC), alcoholic liver disease (ALD) and non-alcoholic steatohepatitis (NASH). Insets show a higher magnification of the parenchymal tissues. Fibrotic septa are delineated by the dashed black lines. Scale bar = 200 µm. Inset scale bar = 50 µm. Surface area quantification of immunohistochemical staining. ****Represents statistical significance where *p* ≤ 0.001. *n* = 4–8 in each group (*bottom right panel*). (**b**) Western blot (*left panel*) and quantification (*right panels*) of the ~180 kDa, ~90 kDa and ~60 kDa species of SCARF-1 in normal liver (NL) and chronic liver disease (CLD). **Represents statistical significance where *p* ≤ 0.01. *n* = 4–8 in each group. Results are regions cropped from the same membrane (see Supplementary Figure 7). (**c**) Sandwich ELISA analysis of soluble SCARF-1 (sSCARF-1) in human serum. *n* = 5–10 in each group. (**d**) Western blot of SCARF-1 in serum from 3 individual PSC patients. Black arrow indicates the major species at ~60 kDa. Results cropped from the same membrane (see Supplementary Figure 7). (**e**) SCARF-1 mRNA expression in normal liver (NL) and chronic liver disease (CLD) tissue. ***Represents *p* ≤ 0.005. *n* = 8 in NL and *n* = 26 in CLD group.

Immunohistochemistry of normal human liver tissue demonstrated localised expression of SCARF-1 within the sinusoids and on the major vasculature of the liver; this was also the case in a range of chronic liver diseases where we noted increased presence of SCARF-1 on vessels and fibrotic septa in chronic inflammatory liver disease (Fig. 1a). Western blot analysis detected a protein of 90 kDa (SCARF-1 predicted size 87.4 kDa) and a dimer species 180 kDa in size that were present in both normal and diseased tissue, in addition to a 60 kDa species present in diseased tissue, which was absent from normal liver tissue (Fig. 1b). Many scavenger receptors are regulated via cell surface cleavage by exofacial proteases resulting in release of a soluble form of the scavenger receptor into the circulation^5^. We confirmed the presence of a soluble form of SCARF-1 (sSCARF-1) in human serum, with values ranging from 1–20 ng/ml in the cohort of samples tested (Fig. 1c) and show via Western blot that the predominant immunoreactive SCARF-1 species in serum is the 60 kDa form (Fig. 1d). However, despite significantly increased expression of the proposed 60 kDa form of SCARF-1 within chronically diseased liver tissue sections (Fig. 1a), there were no significant differences in sSCARF-1 concentrations in serum from patients with chronic liver disease (PSC and PBC) compared to healthy donor controls (Fig. 1c). In contrast to this, increased expression of SCARF-1 mRNA was present in chronic liver disease samples (Fig. 1e).

Patients with chronic liver disease are susceptible to developing hepatocellular carcinoma (HCC) on the background of cirrhosis. Having observed elevated levels of SCARF-1 in end-stage liver disease, we investigated whether SCARF-1 was also expressed in the tumour environment. We detected SCARF-1 in HCC at different stages of differentiation in which the protein and was mainly associated with tumour sinusoids and tumour-associated vessels (Supplementary Figure 1a) and within the tumour margin and on capsule-associated vessels (Supplementary Figure 1b). Staining in poorly differentiated HCC tumour was greatly reduced compared with well- and moderately-differentiated tumours. SCARF-1 mRNA levels in HCC tumour tissue also demonstrated a trend for down-regulation, compared to normal liver tissue (Supplementary Figure 1c).

Given the differential expression of SCARF-1 in normal and diseased livers, we also studied the expression pattern of a close homologue, SCARF-2. Immunohistochemical staining of normal liver demonstrated diffuse cytoplasmic expression of SCARF-2 throughout the parenchymal tissue (Supplementary Figure 2a). In contrast to SCARF-1, SCARF-2 did not appear to be upregulated in disease state, as a similarly diffuse, yet more heterogeneous, cytoplasmic staining pattern was observed in the parenchymal tissue of chronically diseased livers (Supplementary Figure 2a). Expression levels of immunoreactive protein of ~100 kDa in size (predicted molecular weight of SCARF-2, 92 kDa) were similar between normal and diseased tissue (Supplementary Figure 2b). However, consistent with with observations for SCARF-1, we also detected a lower molecular weight species (55 kDa) which was present in diseased tissue, and undetectable in normal liver tissue lysates (Supplementary Figure 2b). This was associated with an upregulation in SCARF-2 mRNA expression (Supplementary Figure 2c).

### HSEC express SCARF-1 *in vivo* and *in vitro*

**Figure 2:**
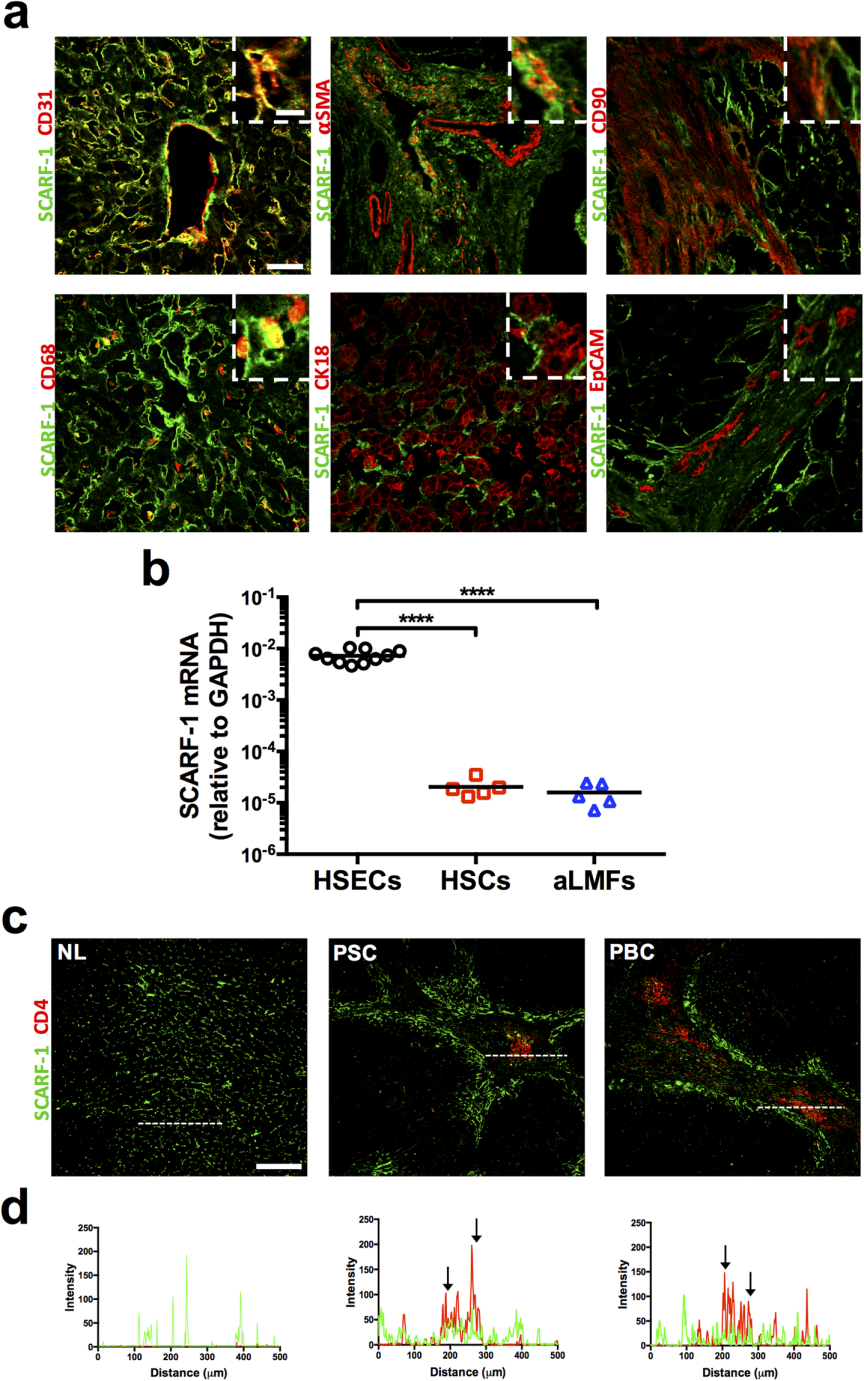
HSEC represent a major cell type expressing SCARF-1 in CLD. (**a**) Representative images of dual colour immunofluorescent staining on chronically diseased (ALD and PSC) liver for SCARF-1 (green) and endothelial marker CD31 (*top left panel*), activated stellate cell marker α-SMA (smooth muscle actin; *top middle panel*), fibroblast marker CD90 (*top right panel*), macrophage marker CD68 (*bottom left panel*), hepatocyte marker CK18 (*bottom middle panel*) and biliary epithelial marker EpCAM (*bottom right panel*). Insets show magnification of SCARF-1 and cell-specific markers. Scale bar = 50 µm. Inset scale bar = 10 µm. (**b**) SCARF-1 mRNA expression in isolated human hepatic sinusoidal endothelial cells (HSECs), hepatic stellate cells (HSCs) and activated liver myofibroblasts (aLMFs). ****Indicates statistical significance where *p* ≤ 0.001. *n* = 5–10 in each group. (**c**) Representative images of dual colour immunofluorescent staining of SCARF-1 (green) and CD4 (red) in normal liver (NL) and chronically diseased livers (PSC and PBC). Scale bar = 250 µm. White dashed lines delineate sites of intensity measurements. (**b**) Intensity measurements of immunofluorescent staining shown in (**a**). Black arrows indicate areas of stronf co-localisation of SCARF-1 (green) and CD4 (red).

Given the differences in expression and distribution of SCARF-1 in chronic liver disease we proceeded to study its cell-specific expression in liver tissue. Dual immunofluorescence staining of CD31 and SCARF-1 within diseased liver tissue demonstrated strong co-localisation within hepatic sinusoidal and vascular endothelium in the liver (Fig. 2a; *top left panel*). Also, co-localisation of SCARF-1 with α-smooth muscle actin (α-SMA) and CD90, markers of activated stellate cells and fibroblasts, respectively, was seen at the interface between the fibrotic septum and parenchymal tissue in chronic liver disease (Fig. 2a; *top middle panel* and *top right panel*). Only a subset of CD68^+^ cells (macrophages) appeared to co-express SCARF-1 (Fig. 2a; *bottom left panel*). Hepatocytes (CK18; Fig. 2a; *bottom middle panel*) and biliary epithelial cells (BEC; EpCAM; Fig. 2a; *bottom right panel*) were negative for SCARF-1 expression.

We next isolated the liver cell types that stained for SCARF-1 in tissue, namely HSEC, hepatic stellate cells (HSCs) and activated liver myofibroblasts (aLMFs). We detected SCARF-1 mRNA in all the cell types but gene expression of SCARF-1 was significantly (300–400-fold) higher in HSEC compared to both HSCs and aLMFs (Fig. 2b). Interestingly, HSEC transcriptional expression of SCARF-1 was also double that of cells from a more conventional endothelium, human umbilical vein endothelial cells (HUVEC; Supplementary Figure 3); the cells in which SCARF-1 was first described^22^.

Our previous work has demonstrated that other scavenger receptors expressed by hepatic endothelial cells, such as stabilin-1, can drive lymphocyte recruitment in chronic liver disease, with particular specificity for regulatory T cells and B cells^15,16^. Consequently, immunofluorescence of liver tissue confirmed that SCARF-1 was prevalent around areas of CD4^+^ T cell infiltration in diseased livers (Fig. 2c,d).

### Regulation of SCARF-1 in HSEC

**Figure 3:**
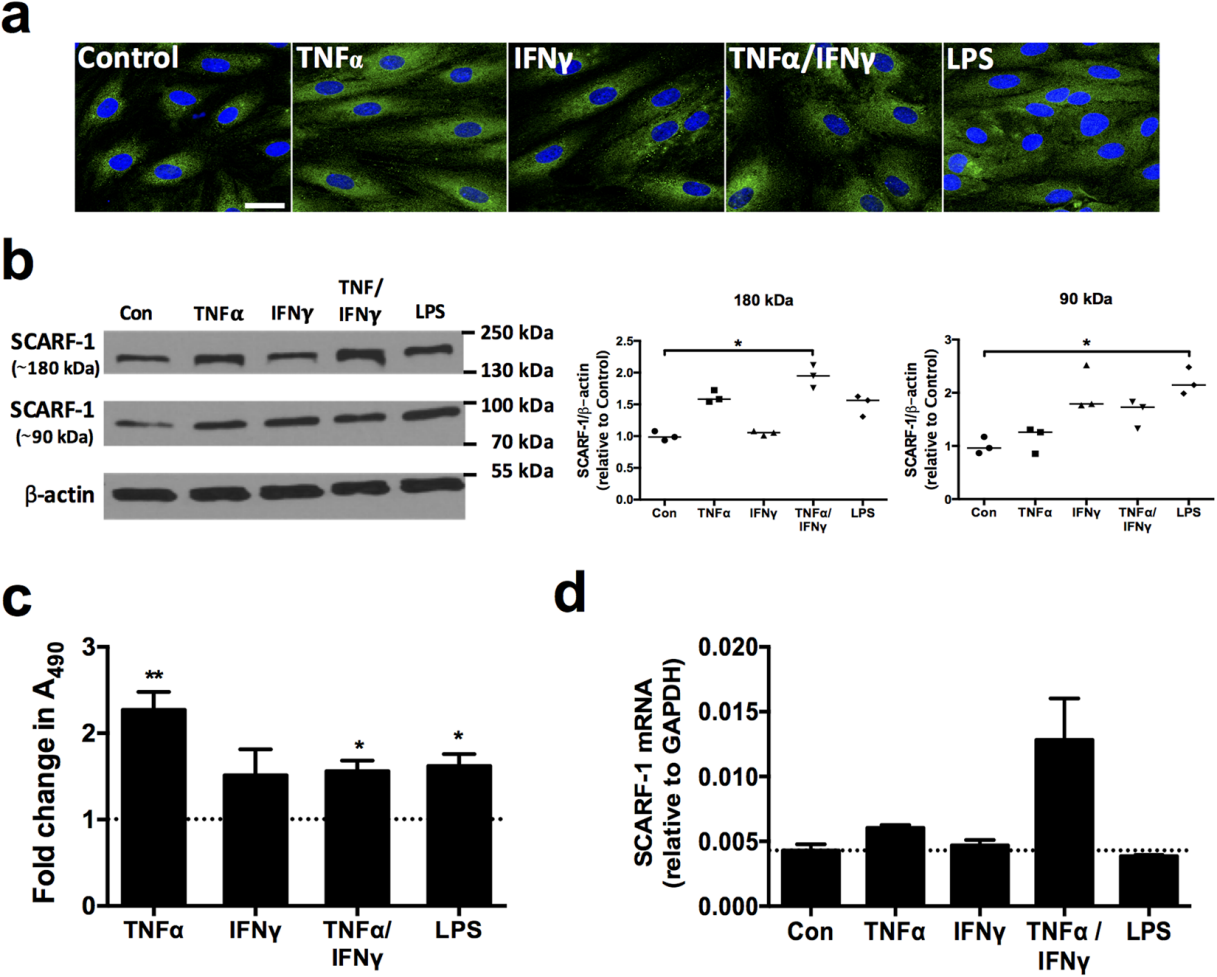
*In vitro* expression of SCARF-1 in HSEC can be up-regulated by proinflammatory cytokines and LPS. (**a**) Representative images of immunofluorescent staining of SCARF-1 (green) with DAPI nuclear stain (blue). Scale bar = 25 µm. (**b**) Representative Western blot (*left panel*) and quantification (*right panels*) of the 180 kDa (dimeric) and 90 kDa (monomeric) species of SCARF-1 in stimulated HSEC compared to media alone control (Con). Results are representative of 3 independent experiments and are regions cropped from the same membrane (see Supplementary Figure 7). (**c**) Fold change in SCARF-1 protein expression measured by cell-based ELISA in stimulated HSEC. (**d**) qPCR analysis of SCARF-1 mRNA in stimulated HSEC. (**a**–**d**) HSEC were treated with 10 ng/ml of tumour necrosis factor (TNF)α, 10 ng/ml of interferon (IFN)γ, or both in combination or with 1 µg/ml of LPS for 24 h. (**c** and **d**) Dotted lines indicate control level of expression. * and ** indicate statistical significance where *p* ≤ 0.05 and *p* ≤ 0.01, respectively. (**b** and **d**) *n* = 3 and (**c**) *n* = 5 independent experiments with different HSEC donors in each group.

We next studied protein expression of SCARF-1 in isolated HSEC, via immunofluorescent staining and found a vesicular and perinuclear distribution of SCARF-1 (Fig. 3a; *far left panel*), which was similar to the intracellular distribution of our previous studies on stabilin-1^15^. Having detected increased SCARF-1 expression in sites of chronic inflammation, we sought to regulate its expression in HSEC with proinflammatory cytokines. TNFα alone and in combination with IFNγ led to an upregulation of SCARF-1 protein as measured by immunofluorescence and cell based ELISA (Fig. 3a; *middle panels*; Fig. 3c). Additionally, we explored the regulatory effects of bacterial lipopolysaccharide (LPS), a known ligand for SCARF-1^33^ and a key factor in many chronic liver diseases^35^ and hepatic malignancies^36^. Incubation of HSEC with LPS led to an increase in SCARF-1 expression, in a similar manner to that observed for cytokine stimulation (Fig. 3a; *far right panel*; Fig. 3c). To confirm these findings we also performed western blotting analysis for protein quantification. We found that in cultured HSEC, SCARF-1 was present in both monomeric and dimeric forms. Increased expression of the dimer was only observed following stimulation with both TNFα and IFNγ whereas increased expression of the monomer was observed following LPS stimulation (Fig. 3b). This effect was largely independent of transcription of SCARF-1 (Fig. 3d). We were unable to detect a 60 kDa immunoreactive species in these cell lysates, possibly reflecting the absence of the myriad proteinases present in chronic liver disease.

Given the high sinusoidal expression of SCARF-1 in HCC tumour, we also investigated the effects of the tumourigenic growth factors, hepatocyte growth factor (HGF) and vascular endothelial growth factor (VEGF) and found that they also increased the cellular expression of SCARF-1 in HSEC, as determined by cell-based ELISA (Supplementary Figure 4a), without increasing transcription (Supplementary Figure 4b). Further analysis of protein expression by Western blotting, once again detected both monomer and dimer forms of SCARF-1 which were relatively unchanged following growth factor stimulation (Supplementary Figure 4c).

### Recombinant SCARF-1 mediates CD4^+^ T cell adhesion

**Figure 4:**
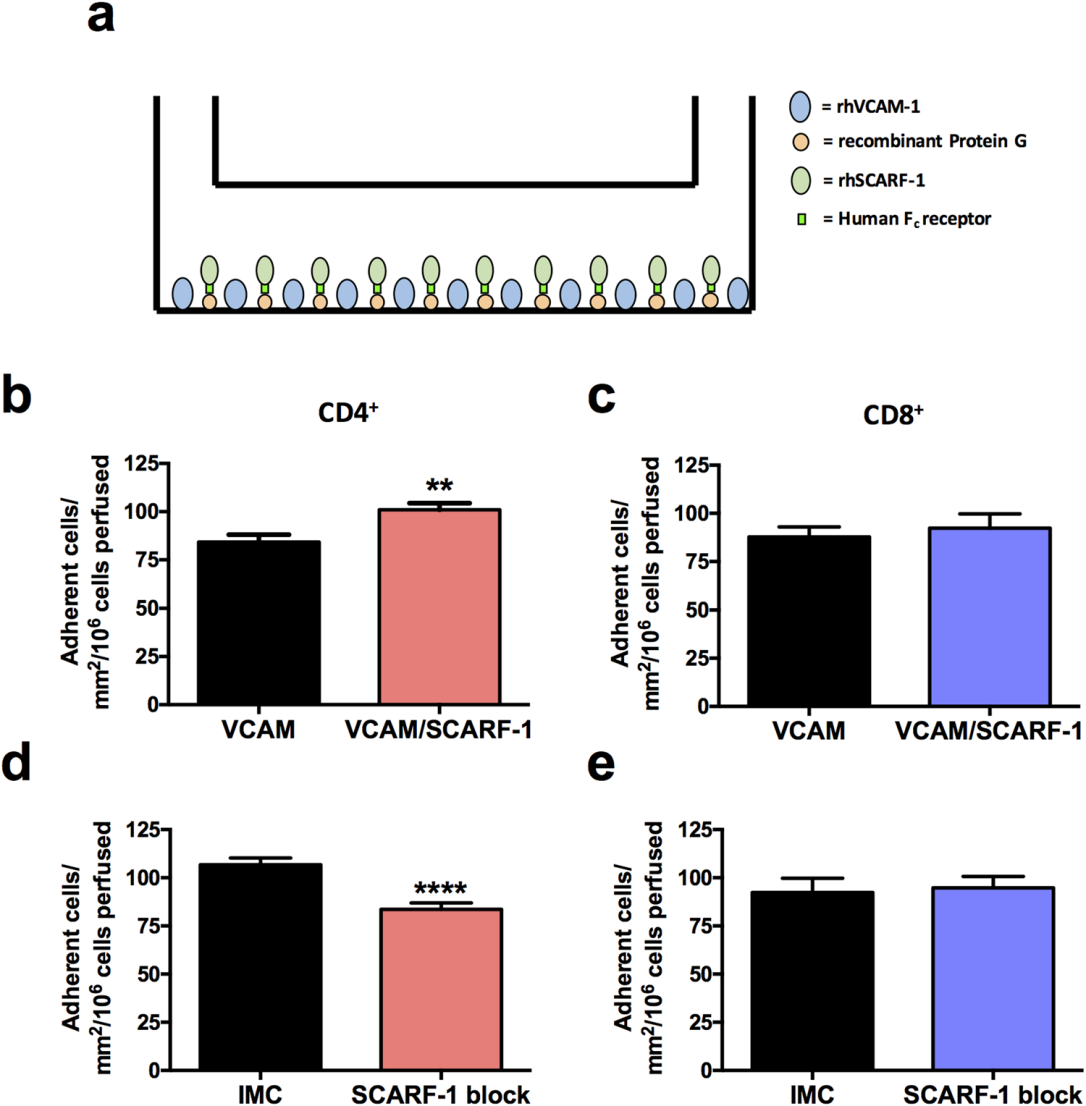
Recombinant human (rh)SCARF-1 mediates the adherence of CD4^+^ T lymphocytes in the presence of rhVCAM-1, under conditions of flow. (**a**) A schematic representation of the immobilised protein flow assay. (**b** and **c**) Quantification of CD4^+^ and CD8^+^ T cells adhered to immobilised rhVCAM-1 (10 µg/ml) and VCAM-1 in the presence of rhSCARF-1 (10 µg/ml). (**d** and **e**) Quantification of CD4^+^ and CD8^+^ T cells adhered to immobilised rhVCAM-1 and rhSCARF-1 pre-treated with isotype matched control (IMC; 10 µg/ml) or SCARF-1 blocking antibody (10 µg/ml). ** and ****indicate statistical significance where *p* ≤ 0.01 and *p* ≤ 0.001, respectively. *n* = 3 independent experiments with different lymphocyte donors, with 12 fields of view taken from each.

Given the similarities in expression of SCARF-1 and stabilin-1, and the fact that both scavenger receptors contain a number of EGF-like moieties in their extracellular domains, we hypothesised that, like stabilin-1, SCARF-1 might function as an atypical adhesion molecule. To test this, we used a flow-based adhesion assay with immobilised recombinant human (rh)SCARF-1 to study interactions of the Jurkat leukaemic T cell line and primary CD4^+^ and CD8^+^ T cells. In order to maintain the correct orientation of the protein in the assay we immobilised a commercially available rhSCARF-1 Fc chimera to plate bound protein G (Fig. 4a). Immobilised rhSCARF-1 alone was unable to support lymphocyte adhesion (data not shown) and because endothelial molecules operate in combinatorial systems we co-immobilised VCAM-1 (which is known to mediate rolling of leukocytes on endothelium^37^) with SCARF-1 and found that the presence of rhSCARF-1 significantly augments the adhesion of Jurkat and CD4^+^ T cells to VCAM-1 under flow conditions (Supplementary Figure 5a and Fig. 4b). Conversely, no effect on CD8^+^ T cell adhesion was detected (Fig. 4c). To confirm that this additional adhesion was a consequence of binding to SCARF-1 and not due to altered stoichiometry of the two immobilised proteins, we were able to reverse the SCARF-1-mediated augmentation of CD4^+^ T cell adhesion with anti-SCARF-1 antibody blockade (Supplementary Figure 5b; Fig. 4d); it should be noted that addition of the SCARF-1 blocking antibody had no effect on CD8^+^ adhesion, thus ruling out non-specific steric interference of antibody binding on T-cell adhesion to VCAM-1 (Fig. 4e). It is also important to note that, in this system, the addition of recombinant protein G alone had no effect on the binding ability of immobilised rhVCAM-1 (data not shown).

### SCARF-1 acts as an adhesion molecule on stimulated HSEC

**Figure 5:**
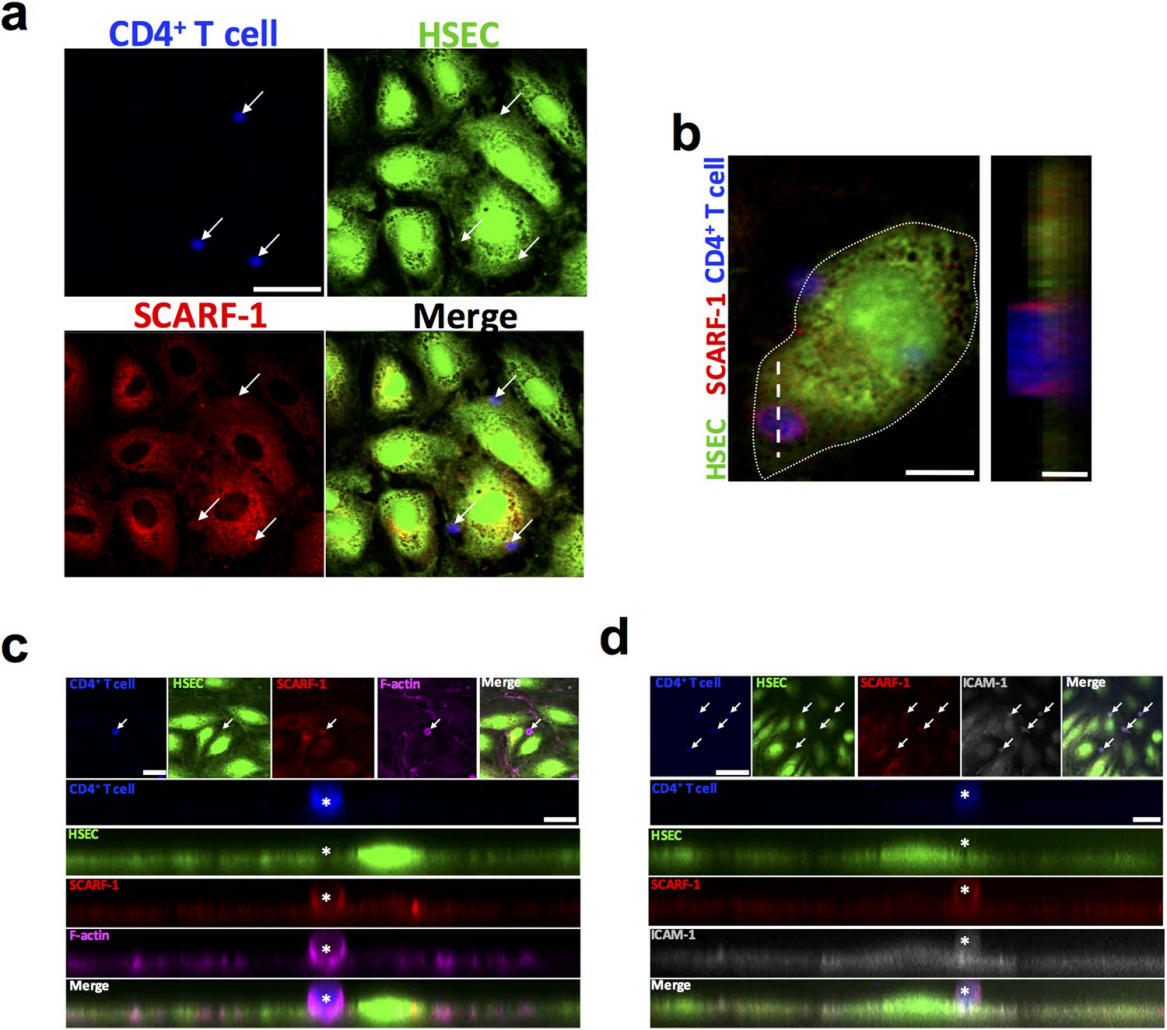
SCARF-1 forms an adhesive cup for CD4^+^ T cell adherence to HSEC. (**a**–**d**) Representative image of CD4^+^ T cells (blue; Cell Trace Violet (CTV)-labelled) attached to TNFα-stimulated HSEC (green; Cell Tracker Green (CTG)-labelled) via SCARF-1 (red) adhesive cups, which are rich in (**c**) filamentous (F-)actin (pink) and (**d**) ICAM-1 (grey). (**a**) White arrows highlight adherent CD4^+^ T lymphocytes. Scale bar = 40 µm. (**b**) White dotted line represents the periphery of the HSEC and the white dashed line delineates the site of the Z stack. Scale bars = 12 µm (*left*) and 2 µm (*right*). (**c**) White arrow highlights adherent CD4^+^ T lymphocyte. Scale bars = 22.5 µm (*top left*) and 7 µm (*right*). (**d**) White arrows highlight adherent CD4^+^ T lymphocytes. Scale bars = 50 µm (*top left*) and 5 µm (*right*). (**c** and **d**) White asterisks indicate adherent CD4^+^ T lymphocyte in adhesive cup.

To understand the relevance of this to HSEC biology we used confocal microscopy to visualise the direct interaction between HSEC-expressed SCARF-1 and CD4^+^ T cells under flow conditions. We found that, where CD4^+^ T cells adhered to HSEC, ring-like clusters of SCARF-1 were evident (Fig. 5a and b; *left panel*). It is important to note that CD4^+^ T cells do not express SCARF-1 mRNA (data not shown) and the SCARF-1 expression pattern observed is thus most likely associated with the endothelial cells and not the lymphocytes themselves. Z-stack imaging confirmed this ring-like enrichment of SCARF-1 present on the HSEC and showed SCARF-1^+^ structures which appear to extend out from the cell surface, thus stabilising the CD4^+^ T cell on the endothelium (Fig. 5b; *right panel*). These structures resembled endothelial docking structures described in previous studies of leukocyte migration^38^. With further immunofluorescent staining we were able to show that SCARF-1+ cups co-localised with filamentous actin and ICAM-1, which are known to be highly expressed in these docking structures (Fig. 5c,d).

**Figure 6:**
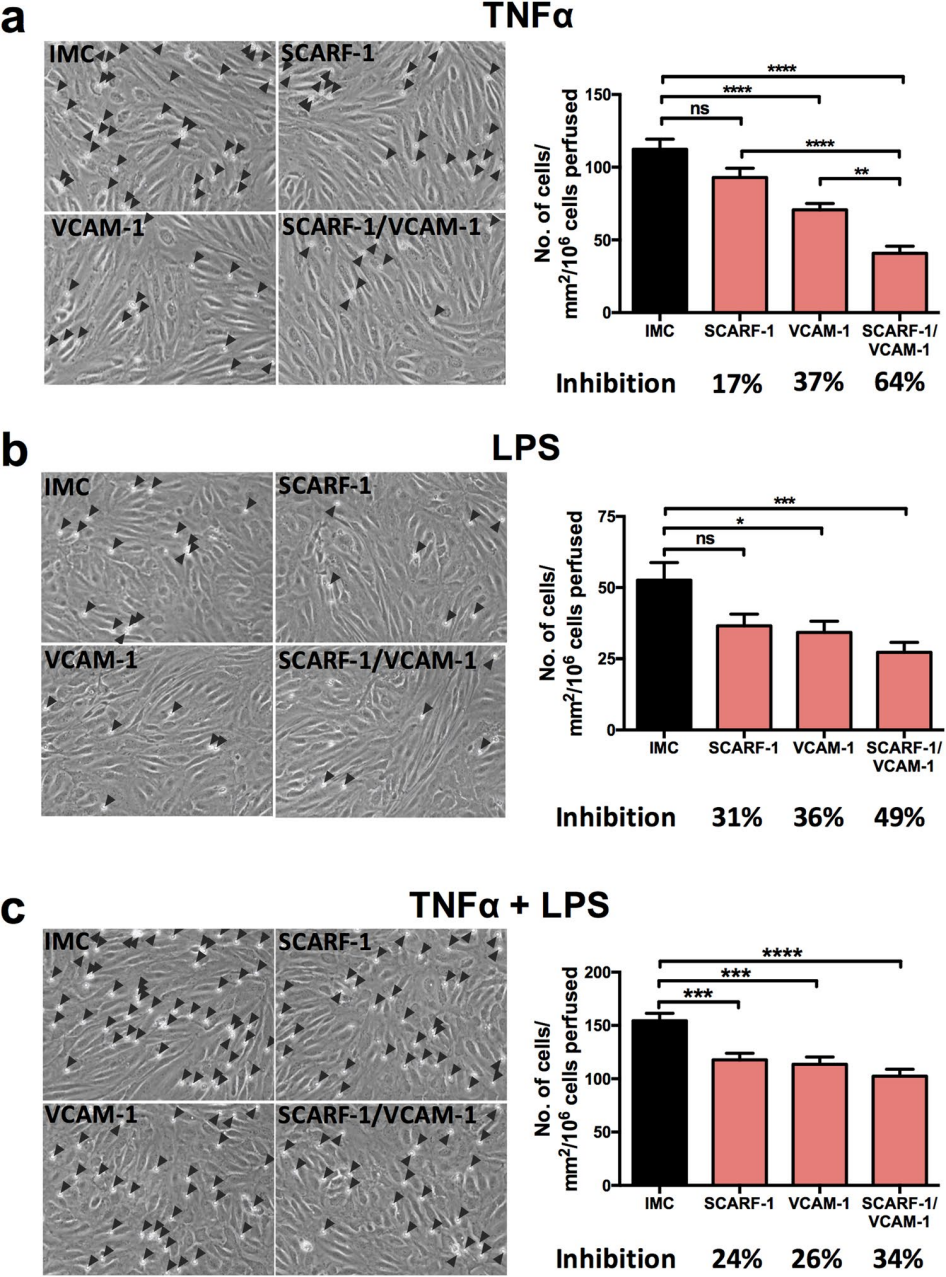
Antibody blockade of SCARF-1 on HSEC inhibits the adherence of CD4^+^ T lymphocytes in the presence of TNFα and LPS. (**a**,**b** and **c**; *left panels*) Representative images of CD4^+^ T cells adhered to HSEC stimulated with (**a**) TNFα (10 ng/ml), (**b**) LPS (1 µg/ml) or (**c**) TNFα and LPS together. HSEC were pre-treated with isotype matched controls (IMC; 10 µg/ml), SCARF-1 blocking antibody (10 µg/ml), VCAM-1 blocking antibody (10 µg/ml) or SCARF-1 and VCAM-1 in combination (both 10 µg/ml). Black arrowheads highlight adherent CD4^+^ T lymphocytes on the HSEC monolayer. (**a**,**b** and **c**; *right panels*) Quantification of adherent CD4^+^ T cells in the presence of the blocking antibodies, with the percentage inhibition indicated. *, **, *** and **** all indicate statistical significance where *p* ≤ 0.05, *p* ≤ 0.01, *p* ≤ 0.005 and *p* ≤ 0.001, respectively. ns = not significant. *n* = 3 independent experiments with different lymphocyte and HSEC donors, with 12 fields of view taken from each.

**Figure 7:**
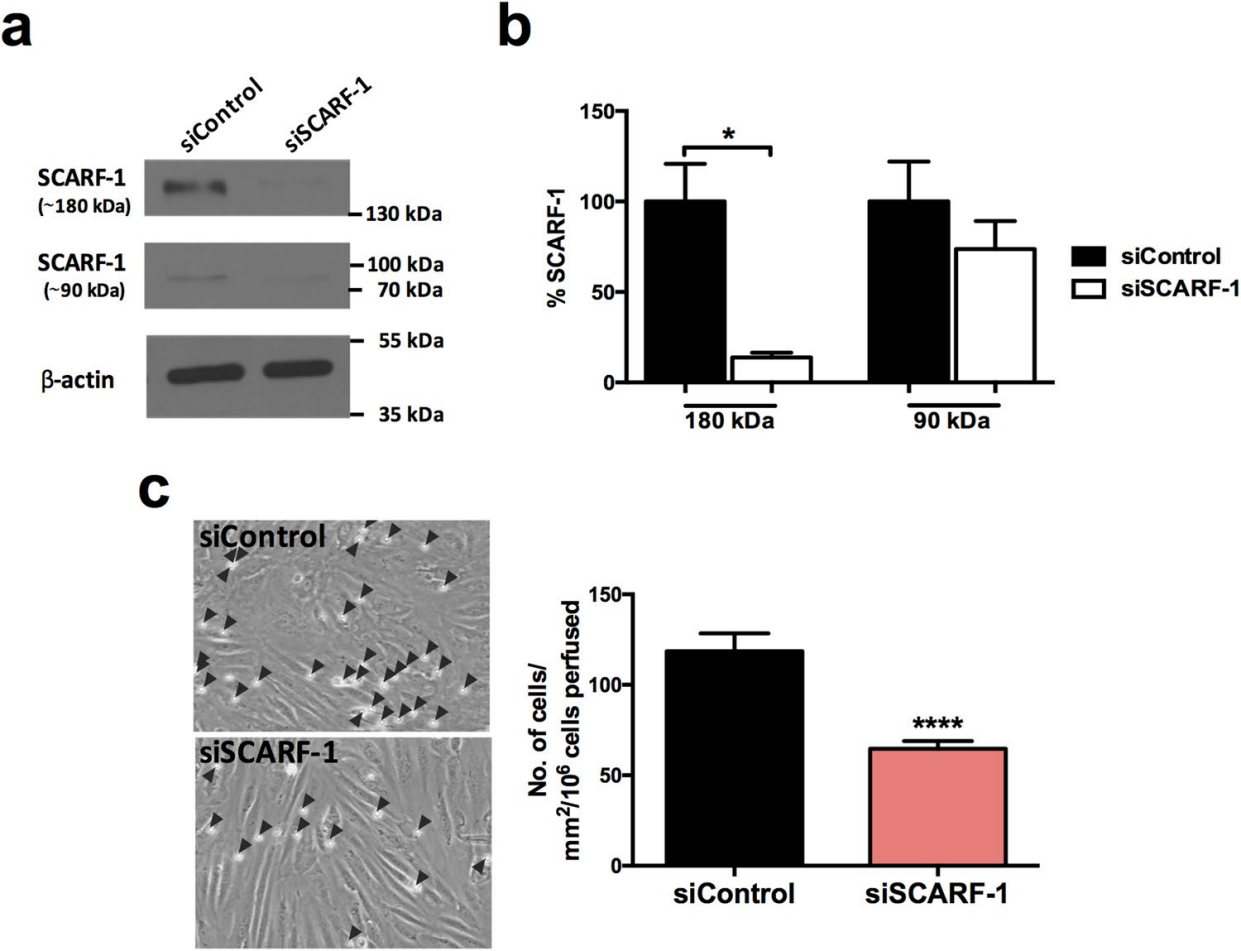
siRNA knockdown of SCARF-1 in HSEC significantly decreases CD4^+^ T cell adherence. (**a**) Representative Western blot analysis of SCARF-1 ~180 kDa and ~90 kDa species in HSEC treated with control and SCARF-1 siRNA knockdown. Results are regions cropped from the same membrane (see Supplementary Figure 7) (**b**) Quantification of SCARF-1 expression in HSEC treated with siRNA knockdown of SCARF-1 expressed as a % of expression in control HSEC. *n* = 3 independent experiments with different HSEC. *Indicates statistical significance where *p* ≤ 0.05. (**c**) Quantification of adherence of lymphocytes to monolayers of HSEC in flow assays pre-treated with control siRNA and SCARF-1 siRNA. *n* = 3 independent experiments with different HSEC donors, with 12 fields of view taken from each. ****Indicates statistical significance where *p* ≤ 0.001.

We next studied the role of SCARF-1 in the adherence of isolated CD4^+^ T lymphocytes to primary HSEC in flow-based adhesion assays. Adhesion of lymphocytes to unstimulated HSEC *in vitro* is very low but can be increased by stimulation with cytokines to induce expression of ICAM-1 and VCAM-1. In order to mimic the pro-inflammatory microenvironment of chronic liver disease as well as gut-derived factors, we used TNFα and LPS to stimulate HSEC before using antibody blockade to investigate the contribution of SCARF-1 to the adhesion process. Under stimulation with TNFα alone, SCARF-1 blockade had a small inhibitory effect (17%) on CD4^+^ T cell adherence to the HSEC, which was approximately half the inhibition seen with an anti-VCAM-1 antibody alone (37%) (Fig. 6a). Combined blockade with both antibodies on the HSEC monolayer had a synergistic effect on inhibition of CD4^+^ T cell adhesion (64%) (Fig. 6a). We confirmed that this phenomenon was CD4^+^ T cell-specific by repeating the flow-based experiments on TNFα-stimulated HSEC with CD8^+^ T cells where SCARF-1 blockade had no effect (Supplementary Figure 6). In HSEC stimulated with LPS, SCARF-1 blockade on CD4^+^ T cell adhesion was more pronounced (31%) and was comparable to that seen with VCAM-1 blockade (36%) (Fig. 6b); nevertheless, the cumulative effect of dual blockade was reduced (49%) (Fig. 6b). Combined HSEC stimulation with TNFα and LPS again showed an effect of SCARF-1 blockade that was comparable to that seen with VCAM-1 alone (24% vs 26%) (Fig. 6c); however, the additive effect of dual blockade was markedly decreased (34%). To confirm the specificity of antibody blockade for SCARF-1 our flow-based adhesion assays were repeated using HSEC which had undergone siRNA-induced knockdown of SCARF-1 expression (confirmed knockdown in three independent isolates) which resulted in a 45% reduction in CD4^+^ T cell adherence (Fig. 7).

## Discussion

Chronic liver disease (CLD) is an increasing global problem and a major cause of morbidity and mortality through the development of fibrosis and cirrhosis which can lead to liver failure or malignancies, such as hepatocellular carcinoma (HCC). Liver injury and fibrosis is driven by uncontrolled immune activation and persistent inflammation, but the factors that regulate this remain poorly understood. The hepatic sinusoidal endothelium is the major route for the recruitment of leukocytes from the blood and plays a major role in regulating liver inflammation through the ability to selectively recruit and activate lymphocyte subsets^39–41^. There is increased interest in the role of gut-derived factors, such as LPS, in regulating liver inflammation and how this interaction of bacterial products with the liver immunity can be targeted for therapeutic benefit^2,42,43^. Here we demonstrate a direct relationship between an LPS-regulated scavenger receptor, SCARF-1, and lymphocyte recruitment. We report for the first time expression of SCARF-1 in human liver tissue and a soluble form in human serum before going on to demonstrate a functional role for SCARF-1 in CD4^+^ T cell adhesion to HSEC. SCARF-1 was strongly expressed on hepatic sinusoids and vessels, primary sites of lymphocyte recruitment during inflammatory liver diseases and a 60 kDa form of the protein was present in chronic liver disease samples, but absent from normal pathological control tissue. SCARF-1 was also present in the sinusoids of hepatocellular carcinoma (HCC) tissue and was observed in tumour-associated and capsule-associated vessels, all of which are important sites for lymphocyte trafficking in HCC.

SCARF-1 expression in HSEC was regulated *in vitro* by proinflammatory cytokines and bacterial LPS; important drivers of chronic liver inflammation. Furthermore, we present evidence that TNFα- and LPS-stimulated up-regulation of SCARF-1 in HSEC leads to a direct interaction with CD4^+^ T lymphocytes. SCARF-1 has previously been shown to play a role in cell-cell adhesion in transfected cell lines^34^, and is able to act as a chaperone molecule in Hsp-mediated antigen cross presentation to both CD4^+^^24^ and CD8^+^^44^ T lymphocytes by antigen presenting cells (APCs); however, to our knowledge this is the first description of SCARF-1 directly contributing to leukocyte recruitment. Taken together, our data suggests that SCARF-1 plays a role in lymphocyte recruitment to hepatic sinusoidal endothelium during inflammatory and bacterial-driven injury of the liver.

Although SCARF-1 expression within the liver was predominantly associated with the sinusoidal and vascular endothelia (Figs 1a and 2a), we also detected SCARF-1 on stromal cells in fibrotic septa *in vivo* and on stellate cells and activated myofibroblasts *in vitro* (Figs 1 and 2a,b). Microbial products that activate TLRs, in particular LPS, can activate hepatic myofibroblasts to produce proinflammatory cytokines and extracellular matrix proteins^35,45^, thus suggesting a potential role for SCARF-1 in fibrogenesis. Furthermore, macrophages play a key role in liver immunology and disease, but we only found SCARF-1 expression on a small subset of liver-resident macrophages *in vivo* (Fig. 2a); consequently, given its more ubiquitous nature and elevated mRNA levels, we concentrated on the expression and function of HSEC-associated SCARF-1. The regulation of SCARF-1 in endothelial cells has only been previously studied at the transcriptional level in HUVEC where proinflammatory cytokines, such as TNFα, IFNγ and IL-1β, were able to inhibit SCARF-1 gene promoter activity^46^. This was highly suggestive of a possible negative feedback loop to limit inflammatory signaling during cytokine stimulation^47^. However, by studying protein expression, we now show that SCARF-1 regulation is potentially more complex. We found that SCARF-1 in liver tissue and primary cultured liver endothelial cells exists in different isoforms. In chronically inflamed liver tissue we found a specific increase in the expression of the 60 kDa form, whereas in isolated HSEC we detected monomeric and dimeric forms of the protein the levels of which were modulated following stimulation with proinflammatory cytokines, LPS or growth factors. Further studies with a NEMO inhibitor demonstrated that SCARF-1 expression in HSEC was not directly related to NF-κB signalling (data not shown), and further studies are required to determine the precise mechanism controlling expression of SCARF-1, as well as the functional role of the soluble form of SCARF-1.

A number of scavenger receptors, such as lectin-like oxidized LDL receptor (LOX)-1^48,49^, stabilin-1^15,16,50^ and stabilin-2^17^, have previously been shown to mediate leukocyte adhesion to endothelial cells. This, added to the fact that SCARF-1 and stabilin-1 both contain a number of EGF-like moieties in their extracellular domains, led us to hypothesise that SCARF-1 could support lymphocyte adhesion to HSEC and thus regulate lymphocyte recruitment to the liver. In flow-based adhesion assay immobilized recombinant SCARF-1 on its own was unable to mediate lymphocyte adhesion. However, when it was co-immobilised with VCAM-1, SCARF-1 augmented the adhesion of Jurkat cells (Supplementary Figure 5a) and primary CD4^+^ T cells under flow conditions (Fig. 4b), whereas it had no effect on CD8^+^ T cells (Fig. 4c), making it one of the few lymphocyte subset-specific adhesion receptors. VCAM-1 has been shown to play an important role in initiating rolling of lymphocytes during recruitment and therefore VCAM-1 is likely to be a pre-requisite for the initial capture of CD4^+^ T cells prior to firm adhesion by SCARF-1. The physiological relevance of our findings was suggested by the subsequent experiments showing that it also mediates CD4^+^ T cell-specific adhesion to primary HSEC under flow particularly to HSEC that had been stimulated with LPS, and TNFα and LPS together. Under these conditions, SCARF-1 showed adhesive properties comparable to the classical adhesion molecule, VCAM-1, and combined blockade of VCAM-1 and SCARF-1 showed an additional reduction of adhesion, thus suggesting that these two receptors could play a combinatorial role in CD4^+^ T cell binding to HSEC (Fig. 6). The identity of the receptor for SCARF-1 present on lymphocytes is unknown, and further studies are required to identify the ligand. We were unable to detect mRNA for SCARF-1 in CD4^+^ T cells, potentially ruling out a homotypic interaction between adjacent SCARF-1 molecules, and we were also unable to detect an effect of SCARF-1 on β1 integrin activation by flow cytometry which suggests that SCARF-1 does not augment binding of T-cells through enhanced integrin/VCAM-1 interactions (data not shown). The fact that SCARF-1 was present in structures surrounding adherent CD4^+^ T cells suggests it may be involved in the organisation of cell membrane structures that promote interactions with CD4^+^ T cells potentially bringing in to play other adhesion receptors or chemokines. Confocal microscopy demonstrated that SCARF-1 co-localised in actin-rich endothelial cups, which also express ICAM-1 (Fig. 5c,d). Subset specificity is unusual in endothelial adhesion receptors, although we have reported such a role for stabilin-1 in CD4^+^ T cell adhesion^15^, and VAP-1 and VCAM-1 have been reported to recruit Th1 and Th2 subsets selectively *in vivo* ^51^.

Our functional assays, together with our findings of constitutive expression of SCARF-1 within normal liver tissue and its up-regulation in HSEC by inflammatory mediators, suggests that SCARF-1 could mediate T cell recruitment during the early inflammatory phase of chronic liver disease pathophysiology. We propose that progressive liver injury promotes the expression of a wide range of proteases, resulting in the cleavage and release of a 60 kDa soluble form of SCARF-1 from the cell surface which then accumulates within chronically diseased tissues. The fact that SCARF-1 is down-regulated in liver cancers, particularly those that are poorly differentiated and thus have the worst prognosis, also suggests it may play a role in tumour immune control through recruitment of inflammatory cells in response to microenvironmental signals. Taken together, its high levels of expression in the liver and the selectivity of its action suggest that SCARF-1 may be an attractive therapeutic target to selectively inhibit liver inflammation without disrupting systemic immune surveillance. SCARF-1 could also represent a new player in the gut-liver axis by regulating CD4^+^ T cell recruitment to liver tissue in response to gut-derived antigens. Several chronic liver diseases are associated with increased gut permeability and bacterial translocation^52^ and, with the onset of these conditions, SCARF-1 may play an important role in the recruitment of inflammatory mediators under these conditions. Further *in vivo* studies are required to confirm the subset specificity and consequences of SCARF-1 blockade, but our results suggest that SCARF-1 could be a new therapeutic target for prevention of these inflammatory conditions.

## Methods

### Human tissue

Human tissue and blood samples were collected from patients admitted to the University Hospitals Birmingham NHS Foundation Trust, with written informed consent and local ethics committee approval; all experiments were performed in accordance with the regulations and guidelines sanctioned by the West Midlands – South Birmingham Research Ethics Committee, Birmingham, UK (LREC reference 06/Q2702/61 and 04/Q2708/41). Normal liver tissue was taken from rejected organ donors deemed unsuitable for transplantation or from resection margins and diseased liver tissue was obtained from patients undergoing transplantation for chronic liver disease or liver malignancies.

### Immunohistochemistry

Immunohistochemistry was performed on 7 μm thick acetone-fixed cryosections. Prior to staining, sections were thawed to room temperature (RT) and hydrated in PBS/0.1% Tween^®^ 20 (PBST) for 5 min. Endogenous peroxidase activity was then blocked with 0.3% hydrogen peroxide in methanol and blocking of non-specific binding was performed by incubation with Casein Solution (Vector Laboratories, Inc.) in 2.5% horse serum (provided with the anti-mouse or anti-rabbit ImmPRESS™ HRP (Peroxidase) secondary antibody kits; Vector Laboratories Inc.). Sections were incubated with anti-SCARF-1 (8 μg/ml; Abcam; ab92308) or anti-SCARF-2 (1.3 μg/ml; Sigma HPA035079) primary antibody diluted in PBS for 1 h at RT and then washed twice in PBST for 5 min. Isotype matched controls at appropriate concentrations were performed in all experiments. Subsequently, sections were incubated with the anti-mouse (SCARF-1) or anti-rabbit (SCARF-2) ImmPRESS™ HRP for 30 min at RT. Excess secondary antibody was washed off with PBST (twice) and sections were then incubated with DAB (Vector Laboratories Inc.) chromogen for 2 min, after which the reaction was stopped with distilled H_2_O. Nuclei were counterstained with Mayer’s Hematoxylin (Pioneer Research Chemicals Ltd.) for 30 s and washed in warm H_2_O for 2 min. Sections were subsequently dehydrated in sequential washes of alcohol^3x^ and xylene^3x^ and mounted using DPX (Phthalate-free) mounting medium (CellPath). Images were taken using an Axioskop 40 microscope (ZEISS) and surface area coverage was calculated using ImageJ software.

### Quantitative real-time PCR

Total RNA was isolated and purified from human liver tissue using the RNeasy^®^ Mini Kit (Qiagen), in conjunction with the RNase-Free DNase Set (Qiagen) and to the manufacturer’s instructions. Approximately 20–30 mg samples of liver tissue were weighed out and then homogenised using gentleMACS™ M tubes (Miltenyi Biotec) and a gentleMACS™ Dissociator (Miltenyi Biotec). Alternatively, HSEC were cultured to confluence in 6-well culture plates (Corning CoStar), stimulated for 24 h (see ‘*HSEC stimulation*’ below), then cells were lysed *in situ* by the addition of RLT buffer. RNA was subsequently isolated using the RNeasy® Micro Kit (Qiagen). Once isolated, RNA quantity and purity was determined using a Nanophotometer™ (Implen GmbH) and reverse transcription of mRNA was performed by the SuperScript^®^III Reverse Transcriptase (Thermo Fisher Scientific). mRNA expression levels of target proteins were assessed by quantitative real-time PCR (qPCR), using predesigned TaqMan^®^ Gene Expression Assays (Applied Biosystems^®^) (Supplementary Table 1) and TaqMan^®^ Universal PCR Master Mix (Applied Biosystems^®^). qPCR was performed on a Roche Lightcycler 480 (Roche) using the following program: 95 °C for 10 min and 45 cycles of 95 °C for 10 s, 60 °C for 1 min, 72 °C for 1 s. Target mRNA levels were normalised to the housekeeping gene and a fold change of relative expression from the appropriate unstimulated control was calculated with the 2^−ΔCt^ and 2^−ΔΔCt^ methods^53^.

### Western blot

Using gentleMACS™ M-tubes (Miltenyi Biotec), ~75 mg of frozen liver tissues were homogenised in CelLytic MT lysis buffer (Sigma-Aldrich) with 1% Protease Inhibitor Cocktail (Sigma-Aldrich), 1% Phosphatase Inhibitor Cocktail 3 (Sigma-Aldrich) and 5 U/ml DNase-I (Sigma-Aldrich). Alternatively, HSEC were cultured to confluence in 6-well culture plates (Corning CoStar), stimulated for 24 h (see ‘*HSEC stimulation*’ below), then cells were lysed *in situ* by the lysis buffer described above. Lysate protein concentrations were determined against a BSA protein standard (Sigma-Aldrich) using the bicinchoninic acid (BCA) assay (Sigma-Aldrich) and following standard protocols. Lysates were normalised to 2 mg/ml and stored at −20 °C.

20 μg of protein lysate was resolved on a 10% SDS-PAGE and subsequently transferred to nitrocellulose membrane (Thermo Fisher Scientific). Membranes were blocked with 5% non-fat milk solution (Marvel) in TBS (SCARF-1) or PBS (SCARF-2) with 0.02% Tween20 (Sigma-Aldrich; TBS/T or PBS/T) for 1 h at room temperature followed by overnight incubation at 4 °C with the primary antibody (SCARF-1, Abcam ab92308, 6 μg/ml or SCARF-2, Sigma HPA035079, 0.8 µg/ml). Following this, membranes were washed 3x in PBS/T and incubated with a horseradish peroxidase (HRP)-conjugated ant-rabbit IgG antibody (Sigma-Aldrich, A0545, 1/ 2500) for 1 h at room temperature. Proteins bands were detected with Enhanced Chemiluminescent Substrate (ECL; Pierce™). Membranes were then stripped with Restore™ Western Stripping Buffer (Thermo Scientific) for 10 mins, washed in PBS/T twice and re-blocked with 5% non-fat milk in PBS/T for 1 h at room temperature and incubated overnight at 4 °C with the housekeeping anti-β-actin antibody (Sigma-Aldrich A5441; 1/2500). Membranes were then washed 3x in PBS/T and incubated with HRP-conjugated ant-mouse IgG antibody (Sigma-Aldrich, A4416, 1/2500) for 1 h at room temperature. Proteins bands were again detected via ECL.

### Sandwich ELISA

Levels of soluble SCARF-1 (sSCARF-1) in healthy donor and diseased human serum were measured via sandwich ELISA using a commercially available kit (Biorbyt) and following the manufacturer’s instructions.

### Immunofluorescence

For immunofluorescent staining, 7 μm acetone-fixed cryosections were thawed and then blocked for non-specific binding by incubation in PBS with 10% goat serum and casein solution, for 30 min at RT. This was followed by 1 h incubation with primary antibodies against the following antigens: SCARF-1 (8 μg/ml, Abcam ab92308); CD31 (5 μg/ml, DAKO JC70A); αSMA (5 μg/ml, Sigma-Aldrich IA4); CD90 (1 μg/ml, eBioscience SE10); CK18 (1 μg/ml, DAKO DC10); EpCAM (5 μg/ml, Progen HEA125). Samples were washed three times in PBS followed by 30 min incubation with Alexa Fluor^®^ conjugated secondary antibodies (1:250; Thermo Fisher Scientific). Nuclei were stained with 300 nM DAPI (Invitrogen) and slides were subsequently mounted with Fluorescence Mounting Medium (DAKO). Fluorescence images were acquired using a Zeiss 780 Zen confocal fluorescence microscope (ZEISS).

### Primary cell isolation and culture

Hepatic sinusoidal endothelial cells (HSEC) were isolated from ∼30 g human liver tissue as described previously^54^. Briefly, tissue was subjected to collagenase digestion (10 mg/ml collagenase IA; Sigma-Aldrich) and was placed on a 33%/77% Percoll (GE Healthcare) density gradient and centrifuged at 800 x *g* for 25 min. The non-parenchymal cell layer was then removed, and the endothelial cells were isolated by positive immunomagnetic selection using CD31 antibody-conjugated Dynabeads (Invitrogen). The endothelial cells were then seeded in rat tail collagen (1 in 100; Sigma-Aldrich)-coated culture vessels in medium composed of human endothelial serum-free media (SFM; Invitrogen) supplemented with 10% human serum (HD Supplies), 10 ng/ml vascular endothelial growth factor (VEGF; PeproTech), and 10 ng/ml hepatocyte growth factor (HGF; PeproTech). Subsequent to the positive selection of HSEC, the residual cells were considered to be a mixed population of activated liver myofibroblasts (aLMFs)^55^ and were cultured in Dulbecco’s Modified Eagle medium (DMEM; Gibco™ by Thermo Fisher Scientific) with 16% FCS. Quiescent hepatic stellate cells (HSCs) were isolated from non-fibrotic liver tissue, as described previously^55,56^. Viability of HSCs was confirmed using trypan blue exclusion, and autofluorescence suggested a purity of >90%. HSCs were cultured in DMEM containing 16% FCS (Gibco™ by Thermo Fisher Scientific) and used within 4 passages. All cell types were grown and maintained at 37 °C in a humidified incubator with 5% CO_2_.

Human umbilical vein endothelial cells (HUVEC) were isolated via a standard protocol^54^ and were cultured on rat-tail collagen coated plastic in human endothelial SFM supplemented with 10% human serum, 10 ng/ml epidermal growth factor (EGF; PeproTech) and 10 µg/ml hydrocortisone (Sigma-Aldrich).

### HSEC stimulation

Prior to stimulation, HSEC were incubated with endothelial SFM with 10% FCS for 24 h. HSEC were then stimulated with either proinflammatory cytokines (tumour necrosis factor (TNF)α and recombinant human interferon (IFN)γ; 10 ng/ml; both from PeproTech), lipopolysaccharide (LPS) from *E*. *coli* 0111:B4 (1 µg/ml; Sigma-Aldrich) or tumourigenic growth factors (VEGF and HGF; 10 ng/ml) for 24 h.

### Immunocytochemistry

For immunofluorescent staining of SCARF-1 in HSEC, cells were cultured to confluence on rat tail collagen (RTC)-coated 8-well glass bottom µ-slides (Ibidi^®^), subsequently stimulated (see ‘*HSEC stimulation*’) and fixed in 4% paraformaldehyde (PFA). Alternatively, HSEC were cultured overnight in RTC-coated μ-Slides VI 0.4 (Ibidi^®^), stimulated for a further 24 h with 10 ng/ml TNFα and had 1 × 10^6^ cells/ml CD4^+^ T lymphocytes perfused over them as described below (see ‘*Flow adhesion assays*’). HSEC and adherent CD4^+^ T cells were then fixed in 4% PFA. Following fixation, all cells were washed in PBS, permeabilised with PBS with 0.3% Tween^®^ 20 for 5 min and then blocked in PBS with 10% goat serum for 20 min. The cells were then incubated at room temperature with anti-SCARF-1 (12 μg/ml; Abcam; ab92308) and anti-ICAM-1 (10 μg/ml; R & D; BBA3) primary antibodies, Alexa Fluor™ 633 Phalloidin (1 in 40; Invitrogen) or IMC antibodies diluted in PBS for 1 h. The cells were then washed with PBS three times and incubated with appropriate Alexa Fluor^®^ conjugated secondary antibodies (1:250; Thermo Fisher Scientific). Nuclei were labeled with 300 nM DAPI. Cells were washed with PBS three times and left in PBS after final wash before imaging on a Zeiss 780 Zen confocal fluorescence microscope (ZEISS).

### Cell-based ELISA

HSEC were grown to confluence in collagen-coated 96-well flat-bottom plates and media was changed to SFM with 10% FCS for 24 h prior to stimulation. Cells were left under basal conditions (Control) or stimulated for 24 h (see ‘*HSEC stimulation*’). Subsequently, cells were fixed in ice cold methanol for 10 mins and washed with PBS twice. Fixed cells were then blocked with 2% goat serum (Sigma-Aldrich) in PBS for 1 h, followed by incubation with primary antibody (5 μg/ml; Abcam ab92308) or IMC control (5 μg/ml; Abcam ab27478) for 45 min at room temperature. The cells were then washed with PBS 1% bovine serum albumin (BSA; Gibco™ by Thermo Fisher Scientific) and incubated with a peroxidase-conjugated goat anti-rabbit secondary Ab (1/500; DAKO P0448) for 45 min at room temperature. The ELISA was developed using *O*-phenylenediamine substrate (OPD; DAKO) according to the manufacturer’s instructions. SCARF-1 in stimulated HSEC was calculated as the mean absorbance from three replicate wells minus the absorbance of an isotype-matched control antibody and expressed as fold change from control (unstimulated) cells.

### Primary lymphocyte isolation

Peripheral blood mononuclear cells (PBMCs) were isolated from whole blood as previously described^54^. Briefly, whole blood was layered onto Lympholyte^®^-H (Cedarlane) and centrifuged at 800 x *g* for 25 min. The PBMC layer was removed and washed once in PBS with 2% FCS and 1 mM EDTA (Gibco™ by Thermo Fisher Scientific) and centrifuged at 800 x *g* for 5 min. A platelet depletion step was then performed by a second wash in PBS with 2% FCS and 1 mM EDTA and centrifugation at 350 x *g* for 10 min. CD4^+^ and CD8^+^ T lymphocytes were isolated from PBMCs by negative selection by the Dynabeads^®^ Untouched™ Human CD4 T Cells Kit and Dynabeads^®^ Untouched™ Human CD8 T Cells Kit, respectively, and in accordance with manufacturer’s instructions.

### siRNA knockdown of SCARF-1 in HSEC

Cells were transiently transfected with 25 nM SCARF1 Silencer^®^ Select siRNA (s16344; Thermo Fisher Scientific) or a non-targeting siRNA control (Silencer^®^ Select Negative Control No. 1 siRNA; Thermo Fisher Scientific), utilising the Lipofectamine RNAiMAX Transfection Reagent (Invitrogen). Briefly, 2.5 × 10^5^ or 7.5 × 10^4^ HSEC were seeded in rat tail collagen-coated 6-well cell culture plates or 0.4 Channel μ-Slides VI (Ibidi^®^), respectively, and cultured overnight. Subsequently, siRNA duplexes diluted in Opti-MEM (Gibco™ by Life Technologies) were mixed with a final concentration of 0.3% Lipofectamine^®^ RNAiMAX and incubated for 10 min at room temperature. Cells were then washed twice with PBS and the duplex/Lipofectamine RNAiMAX mixture was added to the cells and incubated for 4 h at 37 °C. The duplex/Lipofectamine^®^ RNAiMAX mixture was then removed and HSEC medium without antibiotics was added to the cells which were then maintained in standard culture conditions. After 24 h, transfected cells were stimulated with 10 ng/ml TNF-α and incubated for a further 24 h. HSEC cultured in 6-well culture plates were harvested in CelLytic MT lysis buffer with 1% Protease Inhibitor, 1% Phosphatase Inhibitor Cocktail 3 and 5 U/ml DNase-I and SCARF-1 knockdown was confirmed via Western blot analysis (see ‘Western blot’ above). HSEC cultured in 0.4 Channel μ-Slides VI were subsequently used in flow-based adhesion assays (see ‘Flow-based adhesion assays’ below).

### Flow adhesion assays

We used a modified protocol of a flow-based, recombinant protein adhesion assay, previously described by our lab^57^, to determine whether or not SCARF-1 was able to directly interact with primary lymphocytes. Briefly, μ-Slides VI 0.4 (Ibidi^®^) were washed twice with sterile PBS and then incubated with 10 µg/ml recombinant human (rh)VCAM-1 (R & D Systems) and 10 µg/ml recombinant Protein G (Thermo Fisher Scientific) in PBS for 1 h at 37 °C. The slide was again washed twice with PBS and then incubated with 10 µg/ml rhSCARF-1 (R & D Systems) in PBS or PBS alone (VCAM-1 control wells) for 1 h at 37 °C. Slides were washed in PBS twice and blocked with PBS with 10% FCS for 30 min. Finally, antibodies diluted in PBS with 0.1% BSA were added to the slides for 30 min prior to the flow assay. 1 × 10^6^ cells/ml CD4^+^ or CD8^+^ T lymphocytes, in a flow medium of RPMI with 0.1% BSA, were then perfused over the HSEC at a physiological shear of 0.05 Pa. Each channel of the microslide was perfused for 5 min with T lymphocytes, before a 5 min of wash with flow media alone, during which video recordings were taken.

Additionally, flow adhesion assays over monolayers of HSEC, which have also previously been described by our lab^15,58^, were used to investigate the role of SCARF-1 in lymphocyte adhesion to the sinusoidal endothelium, under conditions of physiological flow. Briefly, approx. 7.5 × 10^5^ HSEC were grown to confluence overnight in rat tail collagen-coated μ-Slides VI 0.4. Cells were then stimulated with 10 ng/ml TNFα, 1 µg/ml of LPS or both in combination for 24 h to promote up-regulation of classical adhesion receptors and SCARF-1. 1 × 10^6^ cells/ml CD4^+^ or CD8^+^ T lymphocytes, in a flow medium of Endothelial SFM with 0.1% BSA, were then perfused over the HSEC at a physiological shear of 0.05 Pa. Each channel of the microslide was perfused for 5 min with T lymphocytes, before a 5 min of wash with flow media alone, during which video recordings were taken.

All flow assays were imaged by phase-contrast microscopy with an Olympus IX50 Inverted Microscope (Olympus), with video recordings of 12 frames from each channel taken. Analysis was then performed for quantification of the number of adherent T lymphocytes. The number of adherent cells was then normalised to cells/mm^2^/10^6^ cells perfused using the following equation: (adherent cells/(flow rate (0.28 ml/min)) × bolus (5 min) × field of view area (0.154 mm^2^)) × (1/concentration of lymphocytes (1 × 10^6^ cells/ml)). The addition of blocking antibodies or isotype matched negative control antibodies was performed immediately preceding each assay and incubated for 30 min.

### Statistical analyses

All data were tested for normal distribution by the D’Agostino-Pearson omnibus test. All parametric data are expressed as mean ± standard error of the mean (SEM) and all non-parametric data are expressed as median ± interquartile range (IQR), with the number of experimental repeats (*n*) specified in each case. For single comparisons, statistical significance was determined by unpaired *t*-test (parametric) or Mann-Whitney *U*-test (non-parametric), whereas evaluation of multiple treatments was performed by ANOVA with a *post- hoc* Tukey’s test (parametric) or Kruskall-Wallis one-way analysis of variance with post hoc Dunn’s test (non-parametric). A *p*-value of ≤0.05 was considered as statistically significant. All statistical analyses were undertaken using Prism^®^ 6 software (GraphPad Software Inc.).

### Data availability

The datasets generated during during the current study are available from the corresponding author on reasonable request.

## Electronic supplementary material

Supplementary Figures
