# Supplementary material for: SCARF-1 promotes adhesion of CD4+ T cells to human hepatic sinusoidal endothelium under conditions of shear stress
Source: Sci Rep. 2017 Dec 14;7:17600. doi: 10.1038/s41598-017-17928-4 (PMC5730566; doi:10.1038/s41598-017-17928-4)
Supplement: Supplementary file 1 — Supplementary Figures [file 41598_2017_17928_MOESM1_ESM.pdf]

SCARF-1 promotes adhesion of $CD4^+$ T cells to human hepatic sinusoidal endothelium under conditions of shear stress

Daniel A. Patten[1*], Sivesh K. Kamarajah[1], Joanne M. Rose[1], Joseph Tickle[1], Emma L. Shepherd[1], David H Adams[1], Chris J. Weston[1‡] and Shishir Shetty[1‡]

[1]National Institute for Health Research Birmingham Liver Biomedical Research Unit and Centre for Liver Research, Institute of Immunology and Immunotherapy, University of Birmingham, Birmingham, United Kingdom

[‡]joint senior authors

[*]**Corresponding author**:

Dr Daniel Patten

National Institute for Health Research Birmingham Liver Biomedical Research Unit and Centre for Liver Research, Institute of Immunology and Immunotherapy, University of Birmingham, Birmingham, B15 2TT, United Kingdom

Email: d.a.patten@bham.ac.uk

Tel: +44 121 415 8692

Fax: +44 121 415 8701

Supplementary Table 1 – qPCR primer/probe mixes

Target	Taqman Assay ID
SCARF1	Hs01092477_m1
18S	Hs99999901_s1
GUSB	Hs00939627_m1
GAPDH	Hs99999905_m1

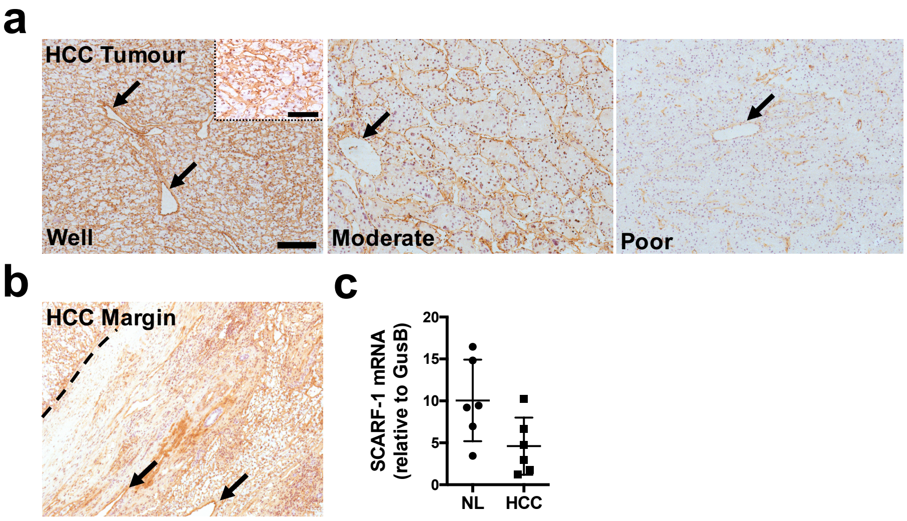

Supplementary Figure 1 – SCARF-1 protein is expressed in hepatocellular carcinoma. (a) Immunohistochemical staining (brown) of SCARF-1 in hepatocellular carcinoma (HCC) tumours of varying differentiation state (well, moderate and poor). Black arrows highlight SCARF-1-expressing tumour-associated vessels. Inset shows a higher magnification of the well-differentiated tumour tissue. Scale bar = 200 μm. Inset scale bar = 50 μm. (b) Immunohistochemical staining (brown) of SCARF-1 in HCC marginal tissue. Black dashed line delineates interface between tumour tissue (left) and capsule (right). Black arrows highlight SCARF-1-expressing capsule-associated vessels. (c) SCARF-1 mRNA expression in normal liver (NL) and HCC tumour tissue. n = 6 in each group.

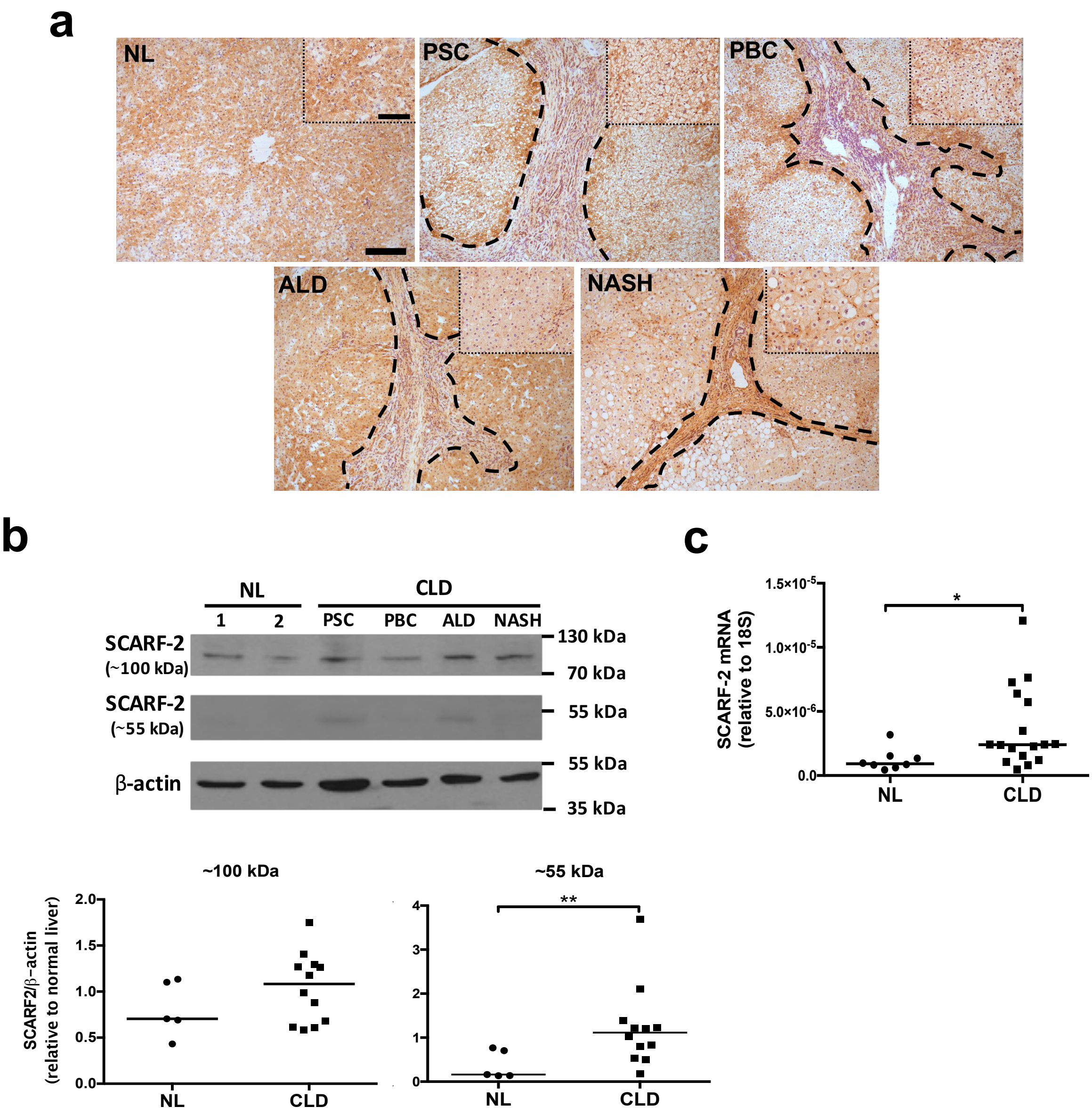

Supplementary Figure 2 – SCARF-2 in normal and chronically liver diseased human liver. (a) Immunohistochemical staining of SCARF-2 (brown) in representative images of normal liver (NL), primary sclerosing cholangitis (PSC), primary biliary cholangitis (PBC), alcoholic liver disease (ALD) and non-alcoholic steatohepatitis (NASH). Insets show a higher magnification of the parenchymal tissues. Fibrotic septa are delineated by the dashed black lines. Scale bar = 200 μm. Inset scale bar = 50 μm. (b) Representative Western blot of SCARF-2 and β-actin protein expression in normal liver (NL) and chronic liver disease (CLD) (*top panel*). Results are regions cropped from the same membrane (see Supplementary

Figure 7). Quantification of ~100 kDa and ~55 kDa SCARF-2 species protein expression, relative to β-actin (*bottom panels*). Data is expressed relative to normal liver (NL). $n = 5$ in NL and $n = 12$ in CLD group. ** represents $p \leq 0.01$ (c) SCARF-1 mRNA expression in normal liver (NL) and chronic liver disease (CLD) tissue. * represents $p \leq 0.05$. $n = 8$ in NL and $n = 17$ in CLD group.

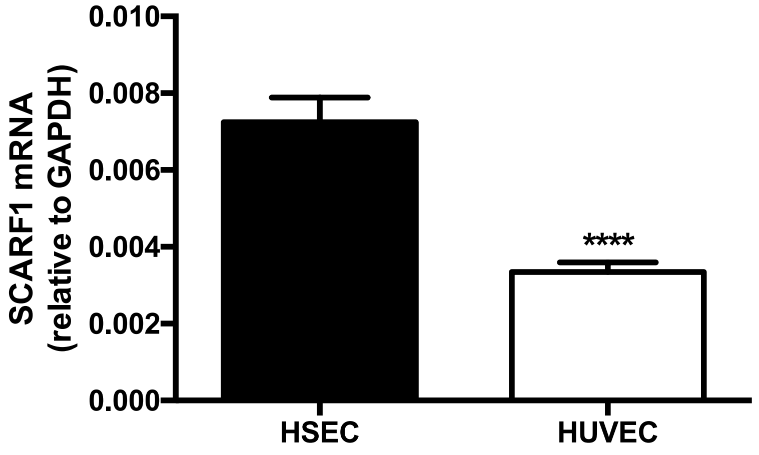

Supplementary Figure 3 – HSEC express significantly higher levels of SCARF-1 mRNA compared to more conventional endothelial cells. HUVEC = human umbilical vein endothelial cells. **** indicates statistical significance where $p \leq 0.001$. $n = 10$ in each group.

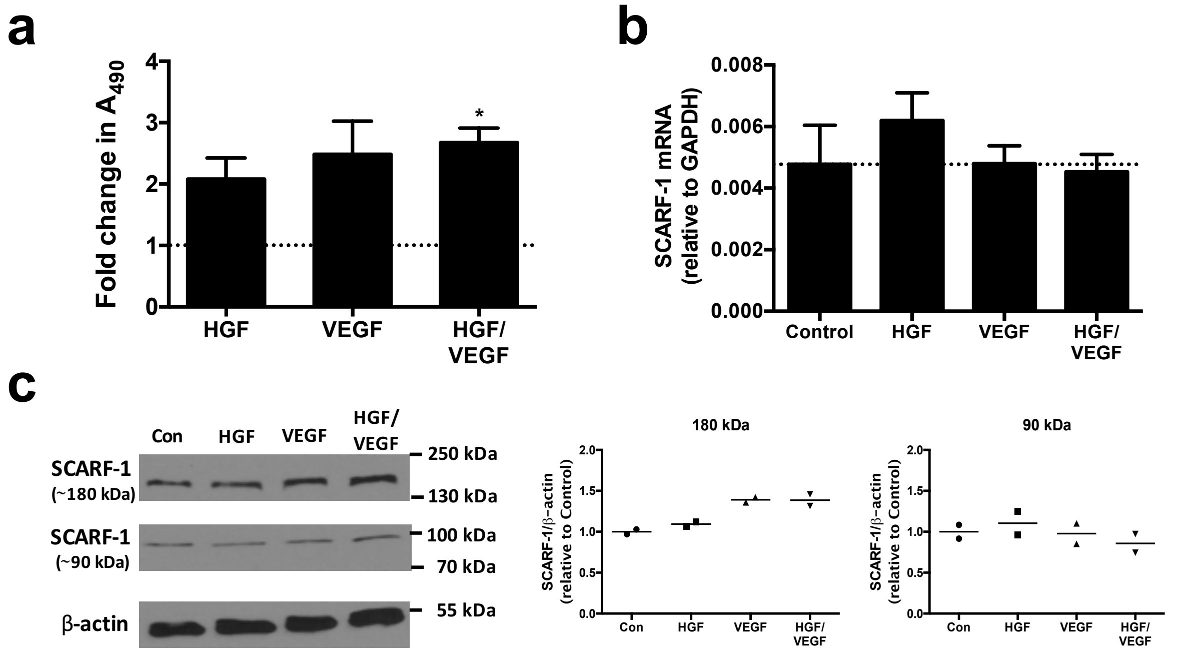

Supplementary Figure 4 - Regulation of SCARF-1 expressed in HSEC by tumourigenic growth factors. SCARF-1 expression in HSEC measured by (a) cell-based ELISA and (b) qPCR. (c) Western blot (*left panel*) and quantification (*right panels*) of the 180 kDa (dimeric) and 90 kDa (monomeric) species of SCARF-1 in HSEC. Results are regions cropped from the same membrane (see Supplementary Figure 7). (a-c) Cells were treated with 10 ng/ml of hepatocyte growth factor, 10 ng/ml vascular endothelial growth factor (VEGF) or both for 24 h. * represents significance from the control where $p \leq 0.05$. (a and b) $n = 3$ independent experiments with different HSEC. (c) $n = 2$ independent experiments with different HSEC.

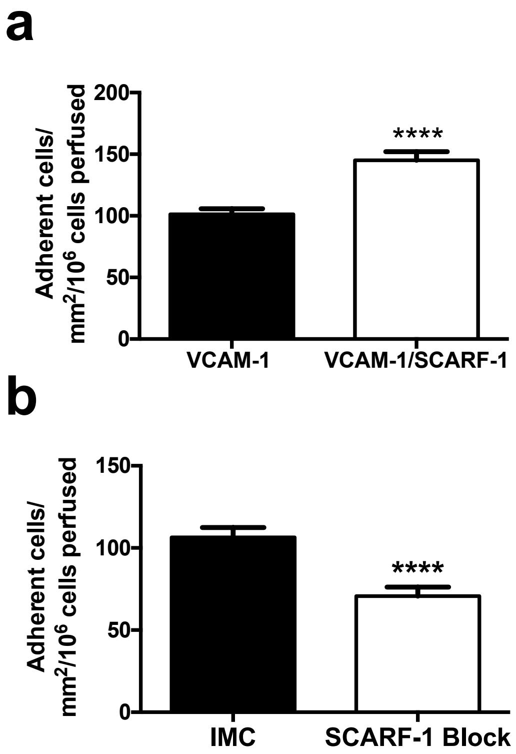

Supplementary Figure 5 - Recombinant human (rh)SCARF-1 mediates the adherence of Jurkat cells in the presence of rhVCAM-1, under conditions of flow. (a) Quantification of Jurkat cells adhered to immobilised rhVCAM-1 (10 μg/ml) and VCAM-1 in the presence of rhSCARF-1 (10 μg/ml). (b) Quantification of Jurkat cells adhered to immobilised rhVCAM-1 and rhSCARF-1 pre-treated with isotype matched control (IMC; 10 μg/ml) or SCARF-1 blocking antibody (10 μg/ml). **** indicates statistical significance where $p \leq 0.001$, respectively. $n = 3$ independent experiments with 12 fields of view taken from each.

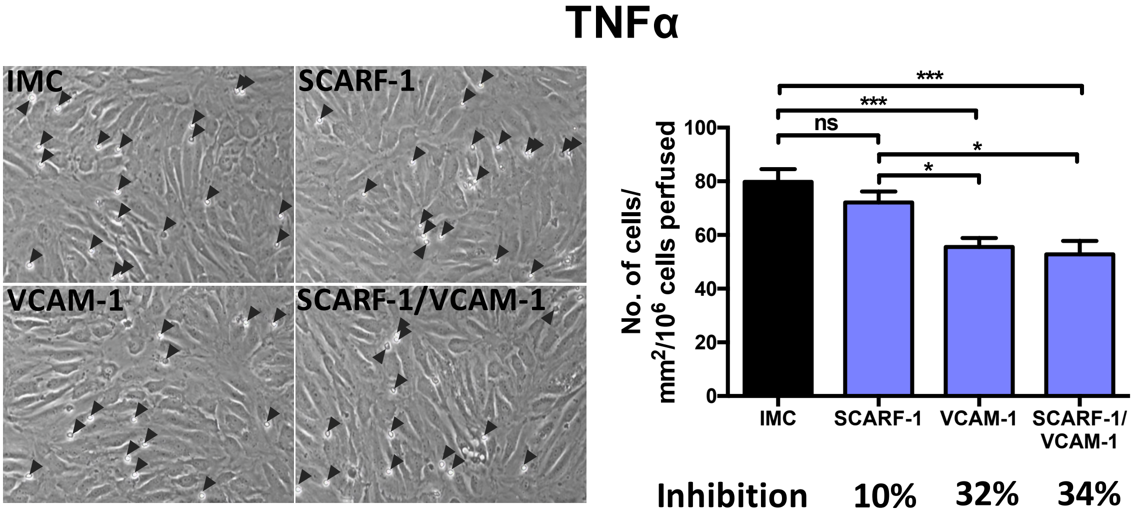

Supplementary Figure 6 – SCARF-1 does not mediate the adhesion of $CD8^+$ T cells to HSEC under flow conditions. (*left panel*) Representative images of $CD8^+$ T cells adhered to HSEC stimulated with TNFα (10 ng/ml). HSEC were pre-treated with isotype matched controls (IMC; 10 μg/ml), SCARF-1 blocking antibody (10 μg/ml), VCAM-1 blocking antibody (10 μg/ml) or SCARF-1 and VCAM-1 in combination (both 10 μg/ml). Black arrowheads highlight adherent $CD8^+$ T lymphocytes on the HSEC monolayer. (*right panel*) Quantification of adherent $CD8^+$ T cells in the presence of the blocking antibodies, with the percentage inhibition indicated. * and *** indicate statistical significance where $p \leq 0.05$ and $p \leq 0.005$, respectively. ns = not significant. $n = 3$ independent experiments with different lymphocyte and HSEC donors, with 12 fields of view taken from each.

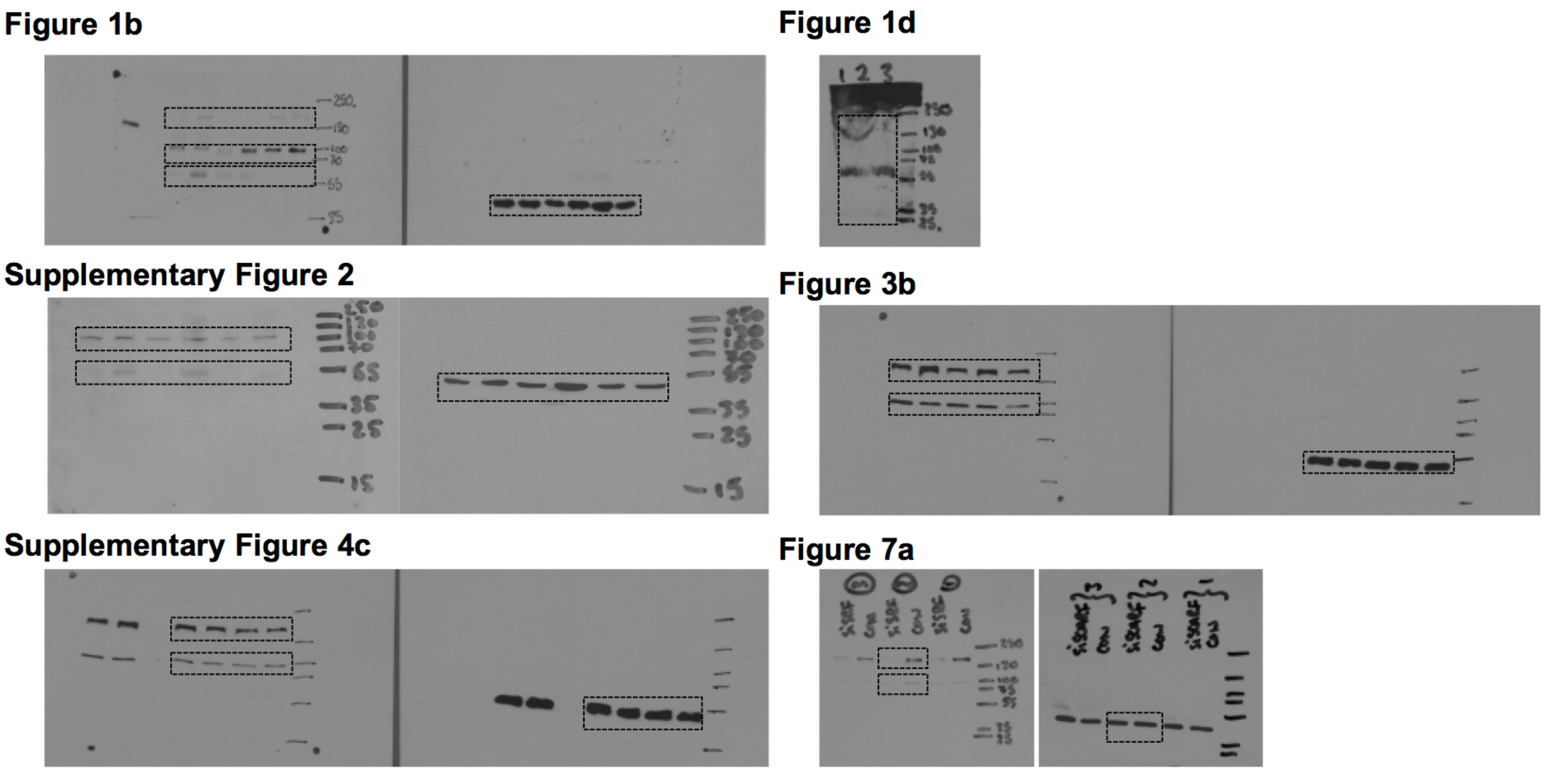

Supplementary Figure 7 – Full scans for all Western blot results. Western blots shown are representative results from multiple experiments performed as described in individual figure captions. Molecular weight markers are indicated on the right-hand side of the blots. The cropped images presented in the respective main Figures are indicated by dashed-line boxes.
